# Abnormal Savda Munziq, an Herbal Preparation of Traditional Uighur Medicine, May Prevent 1,2-dimethylhydrazine-Induced Rat Colon Carcinogenesis

**DOI:** 10.1093/ecam/nep059

**Published:** 2011-06-16

**Authors:** Abdiryim Yusup, Halmurat Upur, Anwar Umar, Benedicte Berke, Dilxat Yimit, Jaya Conser Lapham, Nicholas Moore, Pierrette Cassand

**Affiliations:** ^1^Faculty of Traditional Uighur Medicine, Xinjiang Medical University, 830011 Urumqi, China; ^2^Department of Pharmacognosy, University Victor Segalen Bordeaux 2, 33076 Bordeaux, France; ^3^Laboratory of Food and Colon Carcinogenesis, University Bordeaux 1, 33405 Talence, France

## Abstract

The study tried to assess the chemoprotective effect of abnormal Savda Munziq (ASMq) on 1,2-dimethylhydrazine (DMH)-induced rat colon carcinogenesis. Male F344 rats were randomized into eight groups: Group 1 was served as control, no DMH injection was given and treated daily with normal saline. Rats in Groups 2–8 were given a single intraperitoneal injection of DMH (20 mg/kg body weight) at the beginning of the study. Group 2 was served as negative control, administered with normal saline until the end of the experiment after the single DMH injection. Groups 3–5 were served as pretreatment group, administered with ASMq ethanol extract at 400, 800 and 1600 mg/kg body weight, respectively, until the 45th day, continued by normal saline administration for another 45 days. Groups 6–8 were served as the treatment group, administered with normal saline for the first 45 days from the day of DMH injection, ASMq ethanol extract at three different doses to be administered until the end of the second 45th day. All rats were sacrificed at 91st day and the colons were analyzed for aberrant crypt foci (ACF) formation and crypt multiplicity. Results showed that ASMq ethanol extract reduced the number of ACF, AC and crypt multiplicity significantly (*P* < .05). It suggested that ASMq ethanol extract had chemoprotective effects on DMH-induced colon carcinogenesis, by suppressing the development of preneoplastic lesions, and probably exerted protection against the initiation and promotion steps of colon carcinogenesis.

## 1. Introduction

Abnormal Savda Munziq (ASMq), one of the Uighur medicinal herbal preparations, is widely distributed in the Xinjiang region of China. It has long been used in Traditional Uighur Medicine for the treatment of several diseases such as digestive cancer, diabetes, cardiovascular diseases or chronic asthma [1]. It is regularly used by Uighur physicians for the treatment of cancer, and in southern Xinjiang many ordinary people regularly self-medicate with it to prevent cancers. To date, these putative anticancer effects of ASMq have been little studied and its potential mechanism of action is still unclear. A possible anticancer effect of ASMq clearly warrants further investigation. In our previous studies, ASMq was proved to possess strong free radical scavenging effects, and decreased biological markers of oxidative stress in man [1], protected mitochondria and DNA against OH-induced oxidative damage in a cell-free system [2, 3], inhibited cancer cells proliferation and viability *in vitro* [4].

Colon cancer is one of the most common malignancies in many regions of the world and is thought to arise from the accumulation of mutations in a single epithelial cell of the colon and rectum [6]. Aberrant crypt foci (ACF), a colon carcinoma precursor in humans and rats, is selected as one of the feasible tools and as a sensitive, reliable and rapidly appearing biomarker, supported by the presence of histopathologic intraepithelial neoplasia [7]. Pereira et al. [8] evaluated the ability of a substance to reduce the yield of azoxymethane (AOM)-induced foci in the colon of male Fischer 344 rats as a screening assay for chemopreventive agents, and demonstrated that the ACF might be a useful biomarker to detect possible effects of a chemopreventive agent in rat colon carcinogenesis. In the past several years, a number of chemical carcinogens such as 1,2-dimethylhydrazine (DMH), AOM, 2-amino-3-methyl imidazole quinoline, methylnitrosurea, *N*-methyl-*N*-nitro-*N*-nitrosoguanidine have been used to induce benign and malignant neoplasm in the colon of the rodents. These agents have provided a reasonably accurate experimental model of human colon cancer [9, 10]. One such chemical DMH, a potent and complete carcinogen, has been reliably used to induce initiation and promotion steps of colon carcinogenesis in rodents. DMH and related compounds induce neoplasm specifically in colon of rat even after a single dose [11, 12]. Metabolic activation of DMH to highly reactive electrophiles (methyldiazonium ion) occurs in the liver and colon. However, the main target organ of DMH is the large intestine [9].

As a number of natural compounds prevent colon carcinogenesis in rodents by inhibition of ACF development [13–21], the present study was designed to determine the potential chemopreventive effects of ASMq ethanol extract against the development of colonic ACF in an experimental model of colon carcinogenesis induced by DMH treatment in the rats.

## 2. Material and Methods

### 2.1. Animals

Rats were fed according to ethical standards of animal research committee of the Xinjiang autonomous region. A total of 64 male 6-week-old F344 rats, weighing 180–200 g, were obtained from Harlan (Gannat, France). The animals were kept in polypropylene cages (four animals per cage) on hard wood chips as bedding, and covered with metallic grids in a room maintained at 25 ± 3°C, 50 ± 10% humidity and with a 12-h light–dark cycle. They were fed commercial chow (standard powdered diet for rodent maintenance, 9609 Harlan Teklad, Gannat, France) and provided tap water *ad libitum*.

### 2.2. Chemicals

DMH was obtained from Fluka Sigma-Aldrich (Switzerland), and dissolved in 0.9% NaCl at a concentration of 2 mg/mL and adjusted to pH 6.8 with 1 M NaOH. Formalin solution (10%, neutral buffered) and methylene blue were obtained from Sigma-Aldrich (Lyon, France). All other chemicals were of reagent grade.

### 2.3. Preparation of Ethanol Extract of ASMq

ASMq was composed of 10 kinds of herbal medicinal plants as described in Table 1. Plant materials were purchased from Xinjiang Hospital of Traditional Uighur Medicine (Urumqi 830001, P.R. China). Voucher specimens have been deposited in the Herbarium of Xinjiang Institute of Ecology and Geography, Chinese Academy of Sciences (Urumqi 830011, P.R. China). All herbal materials were authenticated from Xinjiang Institute for Drug Control (Urumqi 830002, P.R. China). Herbal medicines were selected according to the usual recipes recommended by Upur and Yusup [5] and prepared by a professional pharmacist. Briefly, herbs were minced and circulated with 95% ethanol in the proportion of 1 : 10 (w/v) for 5 h and repeated for additional three times for 4 h after adding the same amount of ethanol. The resulting crude extracts were filtered and distilled to oiliness form, and degreased with petroleum ether after dissolving in proper volume of hot water. Remaining water-soluble extract was vacuum dried (Buchi, Switzerland) to powder and kept at 4°C for future use. Extract yield of plant material was 12% (w/w). In this experiment, the dried powder was dissolved in distilled water as a 1600 mg/mL (dry weight of the extract) stock solution and could be kept at −20°C for 3 months without loss of any effect. The other concentrations used in the experiment were prepared by adding distilled water weekly.

### 2.4. Experimental Design

**Figure 1:**
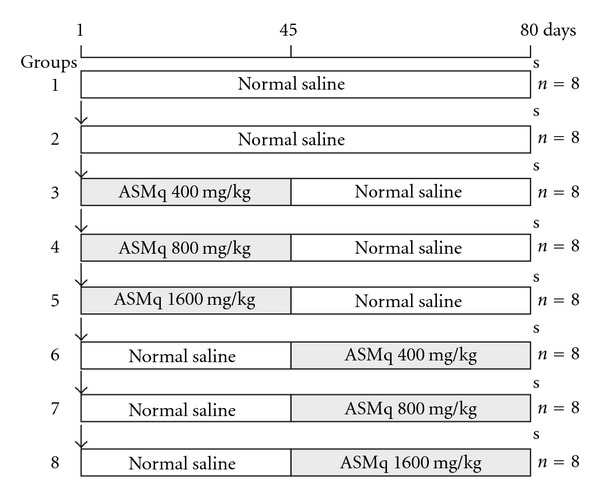
Experimental design to evaluate the effect of ASMq ethanol extract on DMH-induced ACF in the rat colon. Open square: normal saline, yellow square: ASMq administration by gavage, DMH treatment: 20 mg/kg body weight intraperitoneal injection, s: sacrifice at 91st day, *n*: number of animals/group, Group 1: normal control, no treatment, Group 2: negative control, DMH alone, Groups 3–5: pretreatment, ASMq at three different doses (400, 800 and 1600 mg/kg, resp.) and Groups 6–8: treatment, ASMq at three different doses (same as pretreatment group).

After 1 week of acclimatization, animals were randomly divided into eight groups each containing eight rats (Figure 1): Group 1 was served as control, no DMH injection, treated daily with normal saline at 10 mL/kg body weight until the end of the study. Rats in Groups 2–8 were given a single intraperitoneal injection of DMH (20 mg/kg body weight) at the beginning of the study. Rats in Group 2, served as negative control, in addition to DMH injection, were administered normal saline once a day, during the experimental period. Rats in Groups 3–5 served as pretreatment group, ASMq ethanol extract at 400, 800 and 1600 mg/kg body weight were administered by gavage once a day, respectively, after the DMH injection until the end of first 45 days, and then continued by normal saline administration for another 45 days. Rats in groups 6, 7 and 8, served as treatment groups, and administered with normal saline for the first 45 days from the day of DMH injection, subsequently, ASMq ethanol extract at three different doses (400, 800 and 1600 mg/kg body weight, by gavage) were administered, respectively, until the end of second 45 days. The animals were weighed once a week throughout the experimental period, and the dose volume was adjusted once weekly based on body weight. The administration procedure was completed in 90 days after the DMH injection.

### 2.5. Tissue Processing, Identification and Quantification of ACF

Diets were removed 24 h prior to sacrifice, and all rats were sacrificed under ether anesthesia at 91st day after the start of the experiment. The colons were removed, cleaned with saline solution (0.9%) and slit open longitudinally from cecum to anus. Each colon was cut into proximal, middle and distal portion of equal length, and fixed flat between two pieces of filter paper in 10% neutral-buffered formalin for at least 24 h. Later, they were stained with 5% methylene blue for 5–10 min, and they were then placed on a microscopic slide; the mucosal side was observed through light microscope at 40x magnifications. ACF were counted by the method of Bird [22]. Crypts or distinct foci of crypts were counted as an ACF if they displayed at least two of the following characteristics [17]: (i) occupy a greater area than surrounding crypts; (ii) have a thickened epithelial lining; (iii) have elongated or altered shape of luminal opening; and (iv) have an increased pericryptal zone separating the crypt or foci from surrounding crypts. The total number of ACF and the number of crypts/ACF (multiplicity) were scored blindly by a single observer.

### 2.6. Statistical Analysis

All data were presented as the mean ± SD. The statistical analysis was accomplished using SPSS 10.0 software package by one-way analysis of variance (ANOVA) and Student's *t*-test. Differences were considered as statistically significant at *P* < .05.

## 3. Results

### 3.1. Inhibitory Effect of ASMq Ethanol Extracts on Rat Colon ACF, AC Formation

All rats in Groups 2–8 developed colon ACF and AC at the end of the experiment. The number of colon ACF and AC per rat of negative control was 50.3 ± 16.8 and 129.8 ± 43.7, respectively. Administration of ASMq ethanol extracts either pretreatment or treatment caused a significant inhibition of colon ACF and AC formation in a dose-response relationship when compared with negative control group (*P* < .05) (Table 2).

### 3.2. Inhibitory Effect of ASMq Ethanol Extracts on Aberrant Crypt Multiplicity

The number of ACF consisting more than four aberrant crypts per rat of negative control (11 ± 3.9) was significantly higher than those of pretreatment or treatment groups (*P* < .05). As far as aberrant crypt multiplicity (AC/ACF) was concerned, crypt multiplicity of negative control (2.6 ± 0.2) was significantly higher than those of all three pretreatment groups (*P* < .05). Only the high-dose treatment group (1600 mg/kg of ASMq ethanol extract) succeeded to decrease the crypt multiplicity in DMH-treated rats (*P* < .05) (Table 3). A similar tendency was observed for ACF in less than three aberrant crypts/rats.

## 4. Discussion

Biochemical, genetic and morphologic studies have shown that ACF and colon tumors share similar alterations, further supporting the hypothesis that the ACF are precursors of colorectal cancer [23–25]. A number of natural compounds that inhibit ACF development have been proved to prevent colon cancer in rodents [13–21, 26–28]. Thus, the ACF assay, using the complete colon carcinogen DMH, has been proposed to evaluate chemopreventive agents for colon carcinogenesis in rats [29–31]. The purpose of this study was basically to investigate the effect of ASMq ethanol extract on the rat colon carcinogenesis induced by DMH, as evaluated by ACF development.

Recent studies suggested that *in vitro* anticancer properties of ASMq ethanol extract and aqueous extract relate to the inhibition of cell proliferation or cell death triggered by either necrosis (LDH leakage) or apoptosis (caspase-3 activation). Several cellular targets such as cellular macromolecules are impacted by the active molecules. Indeed protein, DNA and RNA synthesis are inhibited [32]. Either related or not a caspase-3-dependent apoptosis is induced, which is also characterized by cell cycle arrest in Sub-G1 phase, downregulation of p53, p21 and bcl2 and upregulation of bax—all shown at transcriptional level in the HepG2 cell line of human liver, which still bears a normal regulating capacity of the *p53* gene. Concomitantly, the herbal preparation strongly prevents the indirect effects of oxidative stress on the DNA such as formation of oxidized DNA bases (8OH-dG) and/or methylated bases such as m5dC. These effects could be regarded as the prevention by ASMq of an epigenetic pathway also leading possibly to cancer [4, 33]. The active ingredients in ASMq that exerted the anticancer effect *in vitro* on Hep-G2 cells have not been fully clarified, but the ethyl acetate extract as well as total flavonoids of ASMq have shown a remarkable cytotoxicity and apoptosis-induction potential in this human hepatoma (HepG2) cell line [34].

Our present results showed that the pretreatment as well as the treatment of ASMq ethanol extract suppressed the development of ACF, AC and crypt multiplicity induced by DMH single injection at all three doses tested. Especially large ACF (four or more) were significantly inhibited after 45 days of pretreatment or treatment with ASMq ethanol extract, since larger ACF (four or more aberrant crypts per focus) have been considered more likely to progress to tumors [35–37]. Our results also showed that the inhibitory effect of ASMq ethanol extract in the treatment group seemed to be more evident than that of pretreatment group. Carcinogenesis is a multistep process, involving initiation, promotion and progression. According to the literature, the increased number of ACF may reflect the initiation step of colorectal carcinogenesis, while the progressive increase in the number of crypts per ACF may correspond to the promotion step of colon tumorigenesis [23, 35, 38–40]. These results indicated that ASMq ethanol extract was able to inhibit the initiation and promotion steps of colon carcinogenesis in rodents.

Other chemopreventive agents with antioxidant properties have been found to inhibit DMH- and AOM-induced colon carcinogenesis and DNA damage in animal model [13, 15, 41, 42]. Although the mechanisms involved in the inhibitory effects of ASMq ethanol extract against ACF formation are not clearly understood, it could be explained by its putative antioxidant activity [43] and protective effects to oxidative stress induced DNA and mitochondrial damage [2, 3]. Since DMH is an indirect carcinogen, which has to be metabolized to exert its carcinogenic effect, it could be postulated that ASMq ethanol extract has interference on DMH metabolic pathways. Also, the presence of dose-response relationship observed in the current study could be attributed to the synergistic activity of congeneric or different compounds increase according to the ASMq ethanol extract dose levels.

In summary, the present study demonstrated the inhibitory effect of ASMq ethanol extract on DMH-induced rat colon carcinogenesis. Of course, as an herbal preparation, ASMq ethanol extract contains a variety of compounds such as alkaloids, flavonoids, polysaccharides, terpenes as well as coumarins (Yusup A, et al., unpublished data), rather than suggesting that chemopreventive action of ASMq ethanol extract may be attributed to the combined effects of various constituents rather than to any single component. Suppression of mutagen-induced oncogenes expression and cell cycle progression and direct modulation of DMH metabolism might be associated to the chemopreventive action of ASMq ethanol extract. Further investigations, including studies elucidating the mechanism involved in its antitumorigenic effects, are warranted to fully evaluate active components for its chemopreventive properties.

## Figures and Tables

**Table 1:** Herbal composition of ASMq [4, 5].

Plant name	Family name	Uighur name	Used parts
*Adiantum capillus-veneris* L.	*Adiantaceae*	Pirsiyavxan	Whole plant
*Alhagi pseudoalhagi* Desv.	*Fabaceae*	Yantak xikiri	Branch secretion
*Anchusa italica* Retz.	*Boraginaceae*	Gavzivan	Aerial parts
*Cordia dichotoma* G.Forst.	*Boraginaceae*	Serpistan	Fruit
*Euphorbia humifusa* Willd.	*Euphorbiaceae*	Xahtere	Whole plant
*Foeniculum vulgare* Mill.	*Apiaceae*	Arpabidiyan	Fruit
*Glycyrrhiza uralensis* Fisch.	*Fabaceae*	Qüqük buya yiltizi	Radix or rhizoma
*Lavandula angustifolia* Mill.	*Lamiaceae*	Üstihuddus	Aerial parts
*Melissa officinalis* L.	*Lamiaceae*	Badrenjibuya hindi	Whole plant
*Ziziphus jujuba* Mill.	*Rhamnaceae*	Qilan	Fruit

**Table 2:** Effect of ASMq ethanol extract on ACF formation in DMH-treated rats (mean ± SD).

Group/treatment	Number of rats	ACF formation in rat colon	
		Total AC	Total ACF

Normal control (NS)	8	0	0
Negative control (DMH + NS)	8	129.8 ± 43.7	50.3 ± 16.8
Pretreatment (DMH + ASMq 400)	8	105.1 ± 60.9*	41.9 ± 20.5*
Pretreatment (DMH + ASMq 800)	8	91.5 ± 37.4*	38.8 ± 14.1*
Pretreatment (DMH + ASMq 1600)	8	53.4 ± 31.8*	23.0 ± 14.0*
Treatment (DMH + ASMq 400)	8	91.8 ± 33.6*	35.9 ± 11.8*
Treatment (DMH + ASMq 800)	8	81.5 ± 39.5*	31.0 ± 14.8*
Treatment (DMH + ASMq 1600)	8	46.0 ± 25.8*	22.4 ± 9.3*

*P < .05 as compared with negative control group.

**Table 3:** Effect of ASMq ethanol extract on aberrant crypt multiplicity in DMH-treated rats (mean ± SD).

Groups/treatment	Number of rats	ACF ≤ 3	ACF ≥ 4	AC/ACF
Normal control (NS)	8	0	0	—
Negative control (DMH + NS)	8	39.3 ± 14.3	11.0 ± 3.9	2.6 ± 0.2
Pretreatment (DMH + ASMq 400)	8	32.9 ± 14.2	9.0 ± 7.3*	2.4 ± 0.4*
Pretreatment (DMH + ASMq 800)	8	32.3 ± 11.2	6.5 ± 3.3*	2.3 ± 0.2*
Pretreatment (DMH + ASMq 1600)	8	19.6 ± 12.5*	3.4 ± 2.9*	2.3 ± 0.5*
Treatment (DMH + ASMq 400)	8	27.8 ± 9.0*	8.1 ± 3.3*	2.5 ± 0.2
Treatment (DMH + ASMq 800)	8	24.6 ± 13.3*	6.4 ± 4.1*	2.6 ± 0.4
Treatment (DMH + ASMq 1600)	8	19.9 ± 7.3*	2.5 ± 2.9*	2.0 ± 0.4*

*P < .05 as compared with negative control group.
